# Leptin potentiates GABAergic synaptic transmission in the developing rodent hippocampus

**DOI:** 10.3389/fncel.2014.00235

**Published:** 2014-08-15

**Authors:** Damien Guimond, Diabe Diabira, Christophe Porcher, Francesca Bader, Nadine Ferrand, Mingyan Zhu, Suzanne M. Appleyard, Gary A. Wayman, Jean-Luc Gaiarsa

**Affiliations:** ^1^Parc Scientifique de Luminy, Aix-Marseille UniversitéMarseille, France; ^2^Unité 901, Institut National de la Santé et de la Recherche MédicaleMarseille, France; ^3^Institut de Neurobiologie de la MéditerranéeMarseille, France; ^4^Program in Neuroscience, Department of Integrative Physiology and Neuroscience, Washington State UniversityPullman, WA, USA

**Keywords:** leptin, GABA, synaptic plasticity, synaptogenesis, hippocampus

## Abstract

It is becoming increasingly clear that leptin is not only a hormone regulating energy homeostasis but also a neurotrophic factor impacting a number of brain regions, including the hippocampus. Although leptin promotes the development of GABAergic transmission in the hypothalamus, little is known about its action on the GABAergic system in the hippocampus. Here we show that leptin modulates GABAergic transmission onto developing CA3 pyramidal cells of newborn rats. Specifically, leptin induces a long-lasting potentiation (LLP-GABA_A_) of miniature GABA_A_ receptor-mediated postsynaptic current (GABA_A_-PSC) frequency. Leptin also increases the amplitude of evoked GABA_A_-PSCs in a subset of neurons along with a decrease in the coefficient of variation and no change in the paired-pulse ratio, pointing to an increased recruitment of functional synapses. Adding pharmacological blockers to the recording pipette showed that the leptin-induced LLP-GABA_A_ requires postsynaptic calcium released from internal stores, as well as postsynaptic MAPK/ERK kinases 1 and/or 2 (MEK1/2), phosphoinositide 3 kinase (PI3K) and calcium-calmodulin kinase kinase (CaMKK). Finally, study of CA3 pyramidal cells in leptin-deficient *ob/ob* mice revealed a reduction in the basal frequency of miniature GABA_A_-PSCs compared to wild type littermates. In addition, presynaptic GAD65 immunostaining was reduced in the CA3 *stratum pyramidale* of mutant animals, both results converging to suggest a decreased number of functional GABAergic synapses in *ob/ob* mice. Overall, these results show that leptin potentiates and promotes the development of GABAergic synaptic transmission in the developing hippocampus likely via an increase in the number of functional synapses, and provide insights into the intracellular pathways mediating this effect. This study further extends the scope of leptin's neurotrophic action to a key regulator of hippocampal development and function, namely GABAergic transmission.

## Introduction

Leptin, the protein product of the *obese* (*ob*) gene is a circulating hormone synthetized predominantly by the adipose tissue, which plays a pivotal role in the control of energy balance through its action on specific hypothalamic nuclei (Zhang et al., 1994; Halaas et al., 1995; Ahima and Flier, 2000). However, accumulating evidence indicates that leptin acts beyond this classical regulatory role and may function as an important developmental signal in the hypothalamus and other brain areas. Indeed leptin receptors (LepR) are widely expressed at early developmental stages throughout the brain, including the neocortex and the hippocampus (Huang et al., 1996; Mercer et al., 1996; Matsuda et al., 1999; Udagawa et al., 2000; Walker et al., 2007; Caron et al., 2010). Further, circulating levels of leptin surge during the early postnatal period and decline to adult levels during the third postnatal week in rodents (Devaskar et al., 1997; Rayner et al., 1997; Ahima et al., 1998). Intriguingly, although it can reach the brain to activate LepRs during the early postnatal period (Carlo et al., 2007; Pan et al., 2008; Caron et al., 2010), leptin administration at this stage has no effect on food intake nor energy expenditure, in sharp contrast to the adult stage (Campfield et al., 1995; Ahima and Flier, 2000; Proulx et al., 2002). Pointing to a neurotrophic role of leptin during development, neuronal projections from the arcuate nucleus of the hypothalamus (ARH) are severely disturbed in leptin deficient (*ob/ob*) mice whereas intra-peritoneal delivery of leptin at early but not adult stages is able to rescue this phenotype (Bouret et al., 2004b; Pinto et al., 2004). Leptin has also been shown to promote neuritic outgrowth in ARH explant cultures (Bouret et al., 2004a, 2012). But beyond its action on the hypothalamus, compelling evidence shows that leptin may have widespread trophic actions and direct the development of extra-hypothalamic structures. Thus, LepR-deficient (*db/db*) mice have a reduced brain weight, a reduced number of cortical neurons, a decreased density in dendritic spines and an immature pattern of synaptic and glial protein expression (Ahima et al., 1999; Udagawa et al., 2006, 2007; Stranahan et al., 2009). Conversely leptin treatment increases brain weight and the number of neuroepithelial cells in *ob/ob* mice, and promotes survival and/or neuritic outgrowth of cerebellar Purkinje cells and cortical neurons in wild type mice (Steppan and Swick, 1999; Udagawa et al., 2006; Valerio et al., 2006; Oldreive et al., 2008). Also of significance, leptin promotes dynamic changes in dendritic morphology and regulates glutamatergic receptor trafficking in the hippocampus, thus modulating the establishment and maturation of glutamatergic synapses (O'Malley et al., 2007; Moult and Harvey, 2009; Moult et al., 2010).

Although the action of leptin on the development and plasticity of glutamatergic synapses has received a lot of attention in the past decade (Shanley et al., 2001; Irving et al., 2006; Oomura et al., 2006; Moult and Harvey, 2009, 2011; for a recent review see Irving and Harvey, 2014), whether and how leptin modulates synapses using γ-amino butyric acid (GABA) as a neurotransmitter has received relatively little attention. Nevertheless, investigating this question is all the more significant as the main effects of leptin on the hypothalamus, including reduction of food intake, increase in energy expenditure and regulation of reproductive function are largely carried out through the modulation of GABAergic transmission (Vong et al., 2011; Xu et al., 2012; Zuure et al., 2013). This observation, in light of the established role of leptin as a neurotrophin, makes it reasonable if not likely that leptin would contribute to the development and plasticity of GABAergic synapses. Consistent with this proposition, circulating levels of leptin surge during the first postnatal weeks of life (Devaskar et al., 1997; Ahima et al., 1998), a developmental period that closely matches the window of GABAergic synaptogenesis (Ben-Ari et al., 2007). Moreover, chronic deficiency of leptin in *ob/ob* mice results in a lower number of GABAergic inhibitory synapses impinging on hypothalamic neuropeptide Y (NPY) neurons (Pinto et al., 2004), while acute application of leptin has been reported to negatively regulate GABAergic synaptic inhibition on hypothalamic POMC neurons (Cowley et al., 2001; Munzberg et al., 2007).

Unraveling the link between leptin and GABA is important also because GABA is a key regulator of brain function and plays a central role in its development (Ben-Ari et al., 2007). GABAergic interneurons regulate neuronal excitability, synaptic integration and network oscillation dynamics and as such are crucial for many cognitive functions. As a result, defective GABA levels and GABAergic transmission are strongly associated with neurodevelopmental disorders and cognitive dysfunction, including major depressive disorder (MDD) (Brambilla et al., 2003; Charych et al., 2009; Deidda et al., 2014). Understanding the factors that modulate the development and efficacy of the GABAergic system is thus of particular interest because it may provide key insights into disease states and potential treatments. Strikingly, leptin levels are also disturbed in several neurological disorders affecting higher brain areas, including MDD. Leptin levels have been reported to be altered in depressed patients while antidepressant treatment has been associated with an increase in leptin levels (Antonijevic et al., 1998; Kraus et al., 2002; Westling et al., 2004; Esel et al., 2005). Animal studies confirmed that a deficiency in leptin signaling is linked with depression and that leptin can act as an antidepressant, possibly via a direct action on the hippocampus (Lu et al., 2006; Signore et al., 2008; Sharma et al., 2010; Yamada et al., 2011; Garza et al., 2012). The fact that MDD is associated with a deficiency in both the leptin and GABA systems thus further supports a functional link between leptin and GABA in extra-hypothalamic areas, particularly the hippocampus. A first illustration of this has been reported in the mature hippocampal network, where authors found acute application of leptin to induce a biphasic modulation of inhibitory GABAergic synaptic transmission (Solovyova et al., 2009).

In the present study we investigated the effect of leptin on developing GABAergic synaptic transmission in the newborn rodent hippocampus, at a time when GABA exerts most of the depolarizing and excitatory drive onto hippocampal neurons (Ben-Ari et al., 2012; Deidda et al., 2014). We show that acute application of leptin results in a calcium-dependent potentiation of miniature GABA_A_ receptor-mediated postsynaptic currents. Pharmacological manipulations point to a postsynaptic signaling pathway involving a cytoplasmic calcium rise from inositol triphosphate (IP_3_)-dependent internal stores, phosphoinositide 3 kinase (PI3K), calcium-calmodulin kinase kinase (CaMKK) and MAPK/ERK kinases 1 and/or 2 (MEK1/2). We also provide evidence that the potentiation of developing GABAergic transmission induced by leptin is expressed as an increase in the number of functional synapses, and that leptin contributes to the development of hippocampal GABAergic synapses *in vivo*.

## Materials and methods

### Animals

All animal procedures were carried out in accordance with the European Union Directive of 22 September 2010 (2010/63/EU). Experiments were performed on both male and female postnatal day 1 (P1) to P7 Wistar rats, and P10 C57BL/6 transgenic mice lacking leptin expression (*ob/ob*, purchased from Charles River Laboratories Italy). Control experiments were performed on wild type (+/+) littermates. Animals were housed in a temperature-controlled environment with a 12 h light/dark cycle and free access to water and food. Mice were genotyped at P6-7 following The Jackson Laboratory genotyping protocol (strain B6.Cg-Lepob/J, ID 000632).

### Acute hippocampal slice preparation

Experiments were performed as previously described (Kuczewski et al., 2008). Brains were removed and immersed into ice-cold (2–4°C) artificial cerebrospinal fluid (ACSF) with the following composition (in mM): 126 NaCl, 3.5 KCl, 2 CaCl_2_, 1.3 MgCl_2_, 1.2 NaH_2_PO_4_, 25 NaHCO_3_, and 11 glucose, pH 7.4 equilibrated with 95% O_2_ and 5% CO_2_. Coronal hippocampal slices (600 μm thick) were cut with a McIlwain tissue chopper (Campden Instruments Ltd.) and kept in ACSF at room temperature (25°C) for at least 1 h before recording. Slices were then transferred to a submerged recording chamber perfused with ACSF (3 mL/min) at 34°C.

### Electrophysiological recordings

Whole cell patch-clamp recordings of CA3 pyramidal neurons (P1-7 rats, P10 mice) were performed with an Axopatch 200B amplifier (Axon Instruments). Microelectrodes (4–7 MΩ) were filled with a recording solution with the following composition (in mM): 110 CsCl, 30 K-gluconate, 10 N-2-hydroxyethylpiperazine-N'-2-ethanesulfonic acid (HEPES), 1.1 ethylene glycol-bis (β-aminoethyl ether)-N,N,N',N'-tetra-acetic acid (EGTA), 0.1 CaCl_2_, 4 MgATP, and 0.3 NaGTP. Series resistance was assessed repetitively in response to a 5 mV pulse and compensated up to 70% throughout the recording. Cells exhibiting more than 20% change in series resistance were excluded from analysis.

Miniature GABA_A_ receptor-mediated postsynaptic currents (mGABA_A_-PSCs) were isolated by adding the voltage-dependent sodium channel blocker tetrodotoxin (TTX 1 μM) and ionotropic glutamatergic receptor antagonists (NBQX 5 μM, D-APV 40 μM) to the perfusion solution, and recorded at a holding potential of −70 mV. In some experiments the identity of miniature PSCs was confirmed by adding gabazine (5 μM) to the perfusion solution at the end of the recording session. Currents were recorded with Axoscope software version 8.1 (Axon Instruments) and analyzed offline with Mini Analysis Program version 6.0 (Synaptosoft). The absence of false positive events was confirmed by visual inspection of each recording. When investigating the effect of postsynaptic loading of pharmacological compounds, compounds were added to the recording solution and allowed to dialyze into recorded neurons for at least 20 min before leptin application. None of the compounds affected the basal frequency or amplitude of mGABA_A_-PSCs during this period. Moreover, the average basal frequency and amplitude of mGABA_A_-PSCs in these neurons were not different than in control neurons.

Peak-scaled analysis of mGABA_A_-PSCs was performed as described by Traynelis et al. (1993) using Mini Analysis program. The absence of correlation between mGABA_A_-PSC decay time course and peak amplitude was verified for each recording. 50–200 events were used for each recording, and events with a decay time distorted by multiple peaks or anomalous noise were eliminated. The variance of individual events was obtained by scaling and subtracting each individual event to the peak of the average event. The mean variance was then plotted against the mean current. The plot was well fitted by a parabolic function yielding the single channel current i_0_ and the number of channels N_0_ contributing to mGABA_A_-PSCs. The single channel conductance γ was calculated from i_0_.

Evoked GABA_A_ receptor-mediated postsynaptic currents (eGABA_A_-PSCs) were isolated by adding ionotropic glutamatergic (NBQX 5 μM, D-APV 40 μM) and GABA_B_ (CGP55845 5 μM) receptor antagonists and recorded at a holding potential of −70 mV. Voltage-dependent sodium channels and the GABA_B_ receptor-activated potassium conductance were blocked in the postsynaptic neuron by adding *N*-(2,6-Dimethylphenyl Carbamoyl Methyl) acetamine (QX314, 50 mM) to the recording solution (Flatman et al., 1982; Andrade, 1991). eGABA_A_-PSCs were generated with pairs of identical stimuli at 50–150 ms interval delivered at a frequency of 0.01 Hz with a bipolar tungsten electrode placed in the CA3 *stratum radiatum*. The paired-pulse ratio (PPR) was calculated as the mean amplitude of the synaptic current evoked by the second stimulus over that evoked by the first one. The coefficient of variation (CV) was calculated as the standard deviation (SD) over the mean of eGABA_A_-PSC amplitude. Average PPR and CV were calculated from 10 to 20 paired stimulations.

### Real-time qRT-PCR

Whole hippocampi (P5 rats) were obtained and treated as described above. RNA was isolated and quantified by reading the absorbance at 260 nm (NanoPhotometer, IMPLEN) using a RNeasy kit (Qiagen), then converted to cDNA using 1 μg RNA and a QuantiTect Reverse Transcription kit (Qiagen) according to the manufacturer's instructions. PCR was carried out with the LightCycler 480 SYBR Green I Master (Roche Applied Science) with 1 μL cDNA using the following oligonucleotides (QuantiTect Primer Assays, Qiagen): leptin receptor (Lepr isoform 1 precursor, QT00182294) and glyceraldehyde-3-phosphate dehydrogenase (GAPDH, QT001199633). Quantitative RT-PCR was performed with a Roche LC480 Light Cycler (Roche Applied Science) following the manufacturer's instructions. Relative mRNA values were calculated using the LC480 software and GAPDH as the housekeeping gene.

### Immunostaining

*pERK immunostaining*. Coronal hippocampal slices (P5 rats) obtained as described above were transferred to the submerged recording chamber and perfused with ACSF supplemented with TTX, NBQX and D-APV for 10 min. Then leptin (100 nM) was added for 15 min or omitted in control experiments. Immediately following treatment slices were fixed overnight at 4°C in phosphate buffered saline (PBS, 0.1 M) with 4% paraformaldehyde (PFA). Coronal hippocampal sections (30 μm thick) were obtained from fixed slices using a cryostat. Nonspecific sites were blocked in PBS with 1% bovine serum albumin (BSA) and cells were permeabilized with 0.3% Triton X-100 detergent (Bio-Rad Laboratories) for 1 h. Sections were then incubated overnight at 4°C with a rabbit phospho-ERK (pERK) primary antibody (1:1000, phospho-p44/42 ERK1/2, Cell Signaling Technology). Sections were rinsed in PBS and incubated for 2 h with a Cy3 donkey anti-rabbit secondary antibody (1:500, Jackson Immunoresearch Laboratories). Hippocampal sections were counterstained for Nissl bodies using the NeuroTrace® green fluorescent Nissl stain (1:1000, Invitrogen), rinsed in PBS and mounted on microscope slides using Vectashield mounting medium (Vector).

*GAD immunostaining*. Under deep anesthesia with chloral hydrate, P14 WT and *ob/ob* mice were intracardially perfused with PBS followed by 4% PFA in PBS. Brains were removed and post-fixed overnight at 4°C. Brains were rinsed in PBS and coronal hippocampal sections (70 μm thick) were obtained using a vibratome (Microm HM 650 V). Sections were incubated first for 1 h in PBS with 1% BSA and 0.3% Triton X-100, then overnight at 4°C with a rabbit glutamic acid decarboxylase 65 (GAD65) primary antibody (1:500, Chemicon AB1511). Sections were rinsed in PBS and incubated for 2 h with an Alexa Fluor 488 donkey anti-rabbit secondary antibody (1:500, FluoProbes). Finally sections were counterstained for Nissl bodies, rinsed in PBS and mounted on microscope slides as described above.

*Data acquisition*. Immunoreactivity was visualized using a laser scanning confocal microscope (LSM 510 Meta, Zeiss) with a 20× air objective and a 63× oil immersion objective. Optical sections were digitized (1024 × 1024 pixels) and processed using ImageJ software (W.S. Rasband, National Institute of Health). Images were thresholded, the area of immunoreactive pixels was measured and expressed as a fraction of the total image area (% area).

### Statistical analyses

Experiments were interleaved in time to ensure homogeneity of experimental conditions. Statistical analyses and assessment of normal distribution were performed with SOFA Statistics software version 1.4.2 (Paton-Simpson & Associates Ltd). A two-tailed, unpaired Student's *t*-test was used to analyze differences between individual groups in mGABA_A_-PSC frequency and amplitude, immunostaining and qRT-PCR datasets. A two-tailed, paired Student's *t*-test was used to analyze differences within one group across conditions, i.e., mGABA_A_-PSC frequency and amplitude and eGABA_A_-PSC amplitude, PPR and CV before (−10–0 min) vs. after leptin application (40–50 min). Sample size for each group reported in electrophysiology experiments is at least 5 cells recorded from at least 4 independent experiments. Sample size for each reported group analyzed using qRT-PCR is three biological replicates. Sample size for each group reported in immunostaining experiments is at least 11 non-overlapping, 150 × 150 μm optical fields from three biological samples. A criterion of p<0.05 was considered to be a “convenient… limit in judging whether a deviation is to be considered significant or not” (Fisher, 1925; Fisher, p. 47). However, acknowledging that “there is no sharp dividing line between probable and improbable results” (Freedman et al., 1991; for a recent review see Hurlbert and Lombardi, 2009), deviations in data were discussed when judged worthy of discussion even if *p* ≥ 0.05. All numerical data are expressed as arithmetic mean ± standard error of the mean (s.e.m.).

### Reagents

The following reagents were purchased from the indicated sources: 1,2,3,4-Tetrahydro-6-nitro-2,3-dioxo-benzo[f]quinoxaline-7-sulfonamide (NBQX), D-2-amino-5-phospho-valeric acid (D-APV) and 3-Aminopropyl(diethoxymethyl)phosphonic acid (CGP55845) from the Molecular, Cellular, and Genomic Neuroscience Research Branch (MCGNRB) of the National Institute of Mental Health (NIMH, Bethesda, MD, USA). STO609, U0126, U73122 and wortmannin from Labnet (Madrid, Spain). Leptin, heparin, isoguvacine, nifedipin, QX314, ruthenium red and SKF96365 from Tocris Cookson (Bristol, UK). Tetrodotoxin (TTX) from Abcam (Bristol, UK). 1.2- bis(2-Aminophenoxy)ethane-N,N,N′,N′-tetraacetic acid (BAPTA) and thapsigargin from Sigma (St Louis, MN, USA).

## Results

### Leptin induces a long-lasting potentiation of miniature GABA_A_ receptor-mediated postsynaptic currents

**Figure 1:**
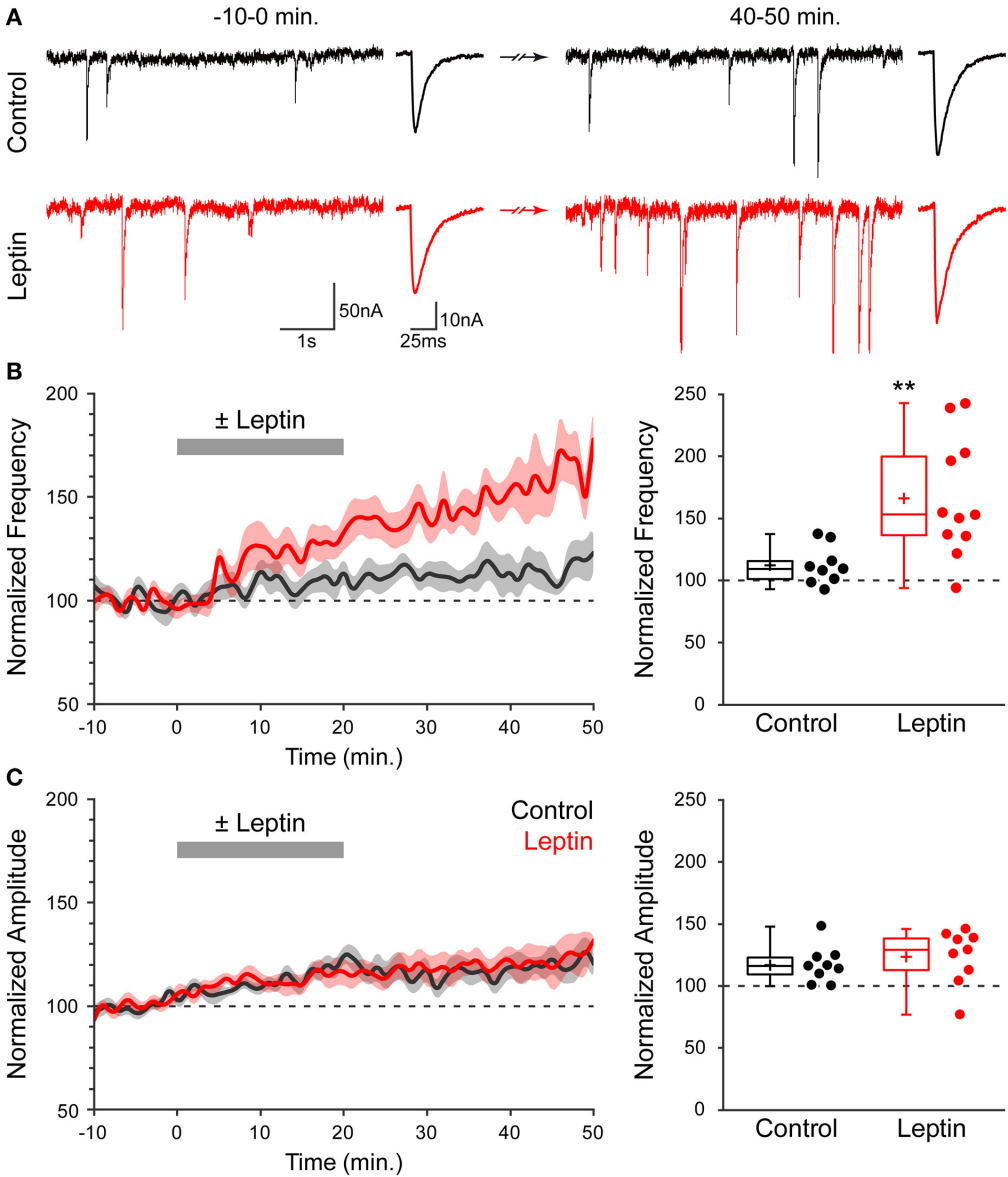
**Leptin induces a long-lasting potentiation of miniature GABAA receptor-mediated postsynaptic currents (mGABA_A_-PSCs)**. **(A)** Acute rat hippocampal slices were made from P1-7 rats. mGABA_A_-PSCs were recorded from CA3 pyramidal neurons in the presence of TTX, NBQX and D-APV. Leptin (100 nM) was applied at *t* = 0–20 min. (red traces) or omitted in Control condition (black traces). Electrophysiological traces of two representative cells show mGABA_A_-PSCs before (*t* = −10–0 min) and after (*t* = 40–50 min) leptin application or during corresponding periods (Control). For each condition: left, recording; right, averaged current. (**B,C**, left) Normalized frequency **(B)** and amplitude **(C)** of mGABA_A_-PSCs against time (1-min bins) showing average value (bold line) ± s.e.m. (shaded area). Plotted are Control (black) and Leptin (red) conditions. Leptin was added from 0 to 20 min (gray bar). (**B,C**, right) Normalized frequency **(B)** and amplitude **(C)** of mGABA_A_-PSCs at the end of the recording (40–50 min). Box plots represent quartiles, whiskers show data range, crosses represent arithmetic mean and scatter plots show individual data points. ^**^p < 0.01 vs. Control (unpaired Student's *t*-test).

We investigated whether leptin induces plasticity of developing GABAergic synapses using acute slices of newborn (P1-P7) rat hippocampi. Miniature GABA_A_ receptor-mediated postsynaptic currents (mGABA_A_-PSCs) were recorded from CA3 pyramidal neurons in the presence of TTX (1 μM), NBQX (5 μM) and D-APV (40 μM), at a holding potential of −70 mV for a minimum of 1 h. Leptin (100 nM) was added to the perfusion solution for 20 min following a baseline recording of at least 10 min; *t* = 0 was defined as the beginning of leptin application. Finally, neurons were recorded for at least 30 min after washout of leptin. We assessed the effect of leptin application on mGABA_A_-PSCs at the end of the recording session (40–50 min) vs. baseline activity (−10–0 min) and vs. interleaved experiments in which leptin was omitted (control experiments). Figure 1A illustrates one typical experiment in which leptin produced a rapid increase in mGABA_A_-PSC frequency within 5–10 min [from 1.86 Hz (−10–0 min) to 3.18 Hz (15–20 min), *p* < 0.05] which persisted for the duration of the recording [3.66 Hz (40–50 min), *p* < 0.05], while no such modification was observed in control condition. A moderate increase in amplitude was also recorded in both cells (Figure 1A). A summary of 20 experiments confirmed that leptin led to a long-lasting potentiation of mGABA_A_-PSC frequency (LLP-GABA_A_) to 166 ± 11% of baseline activity (*n* = 11, *p* = 0.009, Figure 1B, hereafter referred to as “Leptin” condition), while in control condition the frequency of mGABA_A_-PSCs at the end of the recording was modestly increased to 112 ± 5% (*n* = 9, *p* = 0.021, Figure 1B). As a result leptin increased the frequency of mGABAA-PSCs by 1.48-fold compared to control experiments (*p* = 0.005, Figure 1B). On the other hand leptin had no effect on the amplitude of mGABA_A_-PSCs, as the latter was moderately increased after whole cell dialysis for 1 h independently of leptin application (to 117 ± 5% in control vs. 124 ± 7% in Leptin, *p* = 0.084, Figure 1C). The kinetics of mGABA_A_-PSCs were also unaffected by leptin, as the rise and decay times were respectively 1.5 ± 0.1 ms and 11.3 ± 0.7 ms before vs. 1.4 ± 0.1 ms and 11.4 ± 0.7 ms after leptin application (*n* = 11, *p* = 0.5, data not shown). Therefore, exogenous leptin application induces a specific, long-lasting enhancement of miniature GABAergic activity in the newborn rat hippocampus.

**Figure 2:**
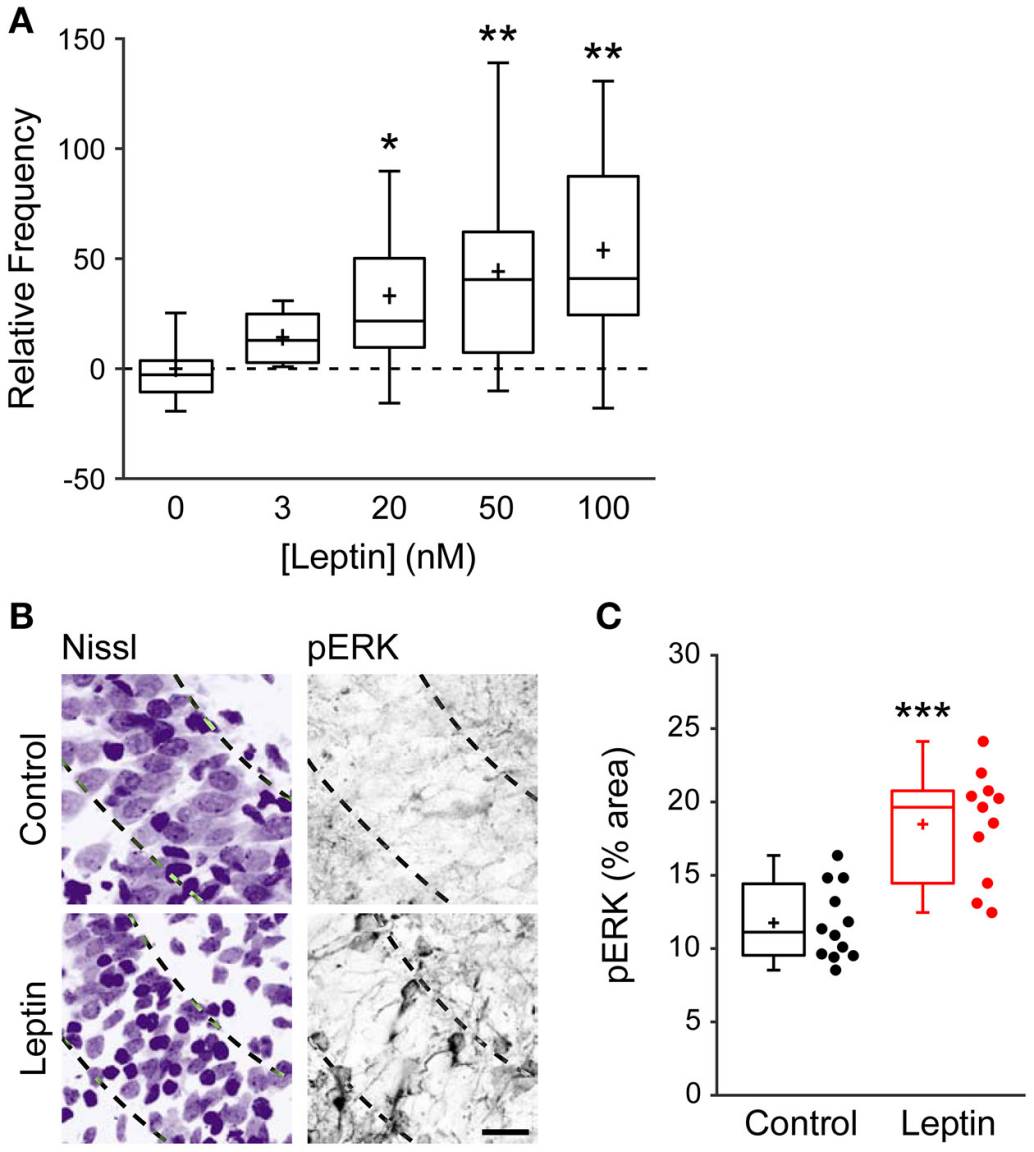
**Leptin exerts a dose-dependent potentiation of mGABA_A_-PSCs and promotes ERK activation**. **(A)** Slices were perfused for 20 min. with various concentrations of leptin. mGABA_A_-PSCs were recorded from CA3 pyramidal neurons, mGABA_A_-PSC frequency was quantified relative to Control condition (Leptin 0 nM) and represented as box plots. ^*^p < 0.05, ^**^p < 0.01 vs. Leptin 0 nM (unpaired Student's *t*-test). **(B,C)** Slices were treated with TTX, NBQX and D-APV and immunostained for phospho-ERK (pERK) following 15 min of leptin treatment (100 nM). Leptin was omitted in Control condition. Representative optical sections **(B)** show somata (Nissl staining, left) and pERK immunoreactivity (right) in CA3 *stratum pyramidale* (dashed lines). For each image: *stratum radiatum*: upper right corner, *stratum oriens*: bottom left corner. Scale bar 25 μm. **(C)** Box plots show pERK immunoreactivity in Control and Leptin conditions. ^***^p < 0.001 vs. Control (unpaired Student's *t*-test).

To narrow down the range of receptors potentially mediating this effect, and because at high concentrations leptin can activate several cytokine receptors, we investigated the action of leptin at lower, nanomolar concentrations (Tartaglia et al., 1995; Devos et al., 1997). Leptin induced a potentiation of miniature GABAergic activity at a concentration of 3 nM and the magnitude of LLP-GABA_A_ gradually increased with leptin concentration to reach a plateau at 50 nM (Figure 2A). Leptin can bind to 6 different isoforms of the leptin receptor (LepR) generated by alternative splicing. Among them, only the long form (LepRb) is able to activate intracellular signaling pathways. Therefore, we probed the presence of LepRb in our conditions using qRT-PCR, which confirmed that the newborn rat hippocampus contains Lepr-b mRNA (Lepr-b/GAPDH mRNA ratio = 1.79 ± 0.35). Finally we investigated whether exogenous leptin application triggers the activation of intracellular pathways in the developing rat hippocampus. Because leptin classically activates the extracellular signal-related kinase (ERK) signaling pathway in the hippocampus (O'Malley et al., 2007; Walker et al., 2007; Caron et al., 2010), we used the activating phosphorylation of ERK as a molecular tool to probe the activation of intracellular pathways by leptin in our system. Thus, we used immunostaining to measure the phosphorylated form of ERK (pERK) in hippocampal slices. Leptin application increased pERK immunoreactivity by 1.46-fold in CA3 *stratum pyramidale* compared to control slices (11.7 ± 0.7% area, *n* = 12 in control vs. 18.48 ± 1.1% area, *n* = 11 in leptin treated slices, *p* < 0.001, Figures 2B,C). Importantly leptin was applied in the presence of TTX, NBQX and D-APV, therefore ruling out a potentially indirect action through network activity. Together, these data strongly suggest that the LepRb is present and functional and show that exogenous leptin application activates intracellular pathways in the newborn rat hippocampus.

### A postsynaptic rise in Ca^2+^ is required for leptin to potentiate miniature GABAergic activity

**Figure 3:**
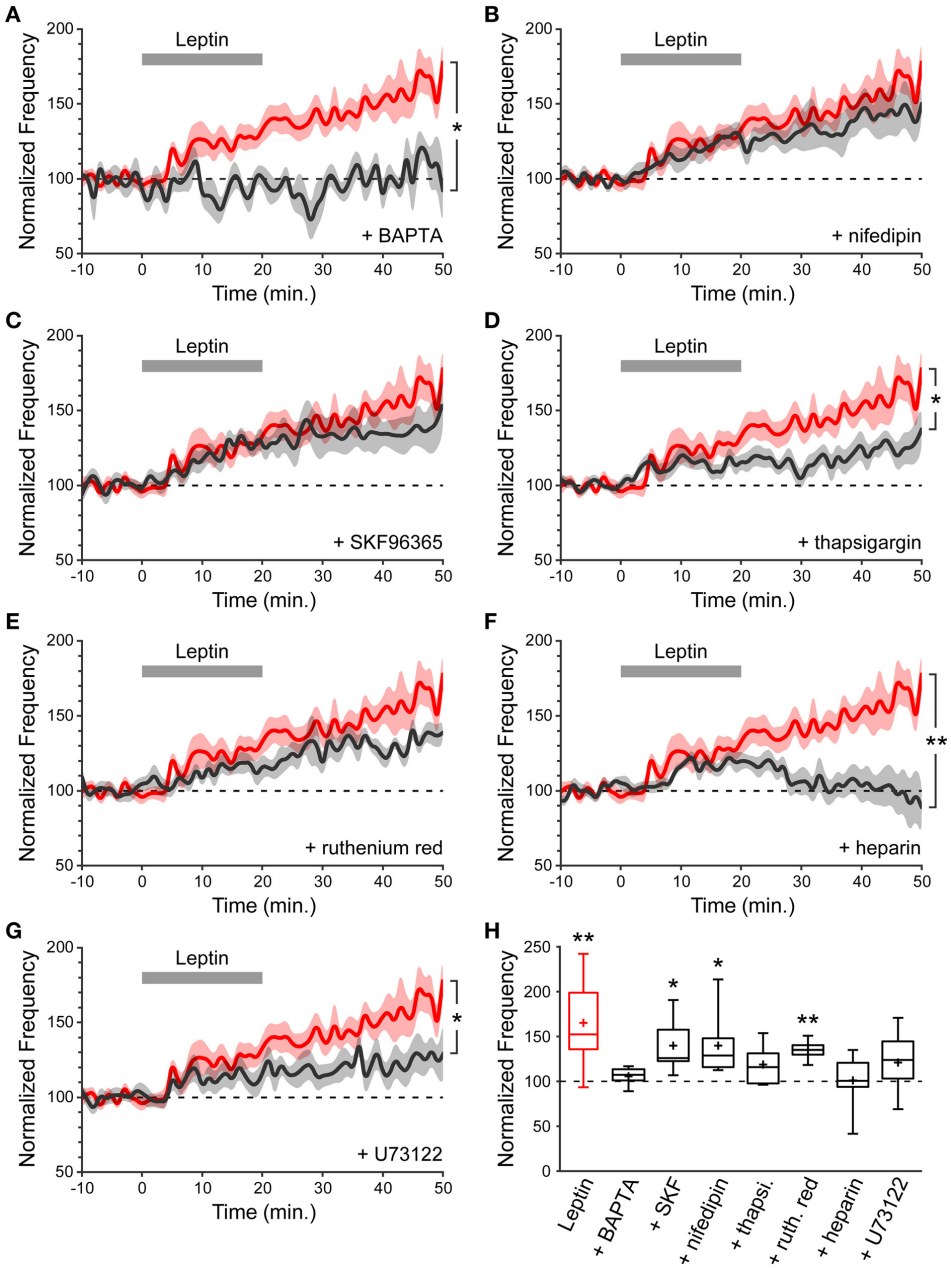
**A postsynaptic rise in Ca2+ is required for leptin to potentiate miniature GABAergic activity. (A–G)** Normalized frequency of mGABA_A_-PSCs against time (1-min bins) showing average value (bold line) ± s.e.m. (shaded area). Leptin was added at *t* = 0–20 min. (gray bar). Plotted are Leptin condition (red) and conditions (black) where leptin was applied in the presence of various pharmacological compounds identified on each panel. Nifedipin and SKF96365 were added in the perfusion solution whereas BAPTA, thapsigargin (thapsi.), heparin, ruthenium red (ruth. red) and U73122 were added in the recording solution. ^*^p < 0.05, ^**^p < 0.01 vs. Leptin, at the end of the recording (40–50 min) (unpaired Student's *t*-test). **(H)** Box plots of normalized mGABA_A_-PSC frequency at the end of the recording (40–50 min). ^*^p < 0.05, ^**^p < 0.01 vs. baseline (−10–0 min) (paired Student's *t*-test).

As a starting point to dissect the signaling cascade activated by leptin to potentiate miniature GABAergic activity, and because a postsynaptic rise in intracellular calcium (Ca^2+^) concentration is a common trigger for long-lasting changes in GABAergic synaptic strength (Gaiarsa et al., 2002), we assessed the contribution of postsynaptic Ca^2+^ to leptin-induced LLP-GABA_A_. To this end we performed whole cell dialysis of recorded neurons with the Ca^2+^ chelator BAPTA (10 mM). BAPTA (and all compounds used hereafter) had no effect *per se* either on the amplitude or frequency of mGABA_A_-PSCs, as assessed by constant baseline values during a dialysis period of at least 20 min before leptin application. We quantified the frequency of mGABA_A_-PSCs at the end of the recording session (40–50 min) and compared it to baseline activity (−10–0 min) and to recordings performed in the absence of other pharmacological compounds (“Leptin” condition). BAPTA completely abolished the leptin-induced LLP-GABA_A_ (106 ± 5%, *n* = 5, *p* = 0.282 and 0.016 vs. baseline and Leptin respectively, Figures 3A,H). Thus, postsynaptic Ca^2+^ contributes to LLP-GABA_A_ induced by leptin.

We then investigated the source of postsynaptic Ca^2+^ recruited by leptin. Several Ca^2+^ sources can contribute to LLP-GABA_A_ induction, including NMDA receptors, voltage-dependent Ca^2+^ channels (VDCCs), canonical transient receptor potential (TRPC) channels and internal Ca^2+^ stores. NMDA receptors were ruled out because the recordings were performed in the presence of D-APV. The contribution of VDCCs is also unlikely because recorded neurons were held at a potential of −70 mV. Nevertheless, we confirmed that L-type VDCCs are not involved by adding nifedipin (10 μM), an L-type VDCC blocker, to the perfusion solution. In the presence of nifedipin leptin could still potentiate the frequency of mGABA_A_-PSCs (140 ± 12% of baseline, *n* = 8, *p* = 0.031 and 0.208 vs. baseline and Leptin resp., Figures 3B,H). Thirdly, leptin was shown to activate a TRPC current in the arcuate nucleus of the hypothalamus and in cultured hippocampal neurons (Qiu et al., 2010, 2011; Dhar et al., 2014a). TRPC channels are non-selective cationic channels permeable to Ca^2+^. We assessed the effect of SKF96365 (50 μM), a commonly used blocker of TRPC channels (Clapham et al., 2005; Rychkov and Barritt, 2007) and found that leptin was still able to induce LLP-GABA_A_ (140 ± 13%, *n* = 6, *p* = 0.021 and 0.252 vs. baseline and Leptin resp., Figure 3C) in the presence of SKF96365 in the perfusion solution.

Finally we investigated the contribution of internal Ca^2+^ stores by depleting them with thapsigargin (3 μM), a non-competitive inhibitor of sarco/endoplasmic reticulum Ca^2+^ ATPases. Thapsigargin prevented the induction of LLP-GABA_A_ (119 ± 8%, *n* = 8, *p* = 0.169 and 0.019 vs. baseline and Leptin resp., Figures 3D,H), indicating that postsynaptic internal Ca^2+^ stores may contribute to leptin-induced LLP-GABA_A_. In a following set of experiments, ruthenium red (20 μM), a blocker of ryanodine receptors (RyRs, Ca^2+^-induced Ca^2+^ release) or heparin (2 mg/mL), a blocker of inositol triphosphate receptors (IP_3_Rs, IP_3_-induced Ca^2+^ release) were added to the recording solution to identify the trigger for postsynaptic Ca^2+^ release from internal stores following leptin application. Whole cell dialysis with ruthenium red failed to prevent LLP-GABA_A_ (135 ± 5%, *n* = 6, *p* = 0.006, and 0.141 vs. baseline and Leptin resp., Figures 3E,H) whereas the potentiation of mGABA_A_-PSC frequency was fully abolished in the presence of heparin (101 ± 12%, *n* = 7, *p* = 0.477, and 0.006 vs. baseline and Leptin resp., Figures 3F,H), pointing to the involvement of postsynaptic IP_3_-induced Ca^2+^ release in the leptin-induced LLP-GABA_A_. Finally, speculating an increased production of IP_3_ by phospholipase C gamma (PLCγ) we added U73122 (5 μM), an inhibitor of PLCγ, to the recording solution and observed a reduction of LLP-GABA_A_ (121 ± 7%, *n* = 11, *p* = 0.121, and 0.020 vs. baseline and Leptin resp., Figures 3G,H).

Taken together, these data indicate that the potentiation of miniature GABAergic activity induced by leptin requires a postsynaptic rise in cytoplasmic Ca^2+^ released from internal stores, through an IP_3_-dependent mechanism.

### A postsynaptic pathway involving ERK, PI3K and CaMKK mediates the leptin-induced potentiation of miniature GABAergic activity

We next investigated the downstream pathway activated by leptin. Leptin acts via multiple intracellular signaling pathways including signal transducer and activator of transcription 3 (STAT3), extracellular signal-related kinase (ERK) and phosphoinositol-3 kinase (PI3K) (Ahima and Osei, 2004; Frühbeck, 2006). Hippocampal neurons do not exhibit increased pSTAT3-immunoreactivity after peripheral leptin injection *in vivo* or leptin treatment of neuronal cultures (Walker et al., 2007; Caron et al., 2010; Dhar et al., 2014b), suggesting that leptin could activate alternative signaling pathways such as ERK or PI3K. Indeed leptin modulates the efficacy of hippocampal glutamatergic synaptic transmission through the activation of PI3K- and/or ERK-dependent pathways (Shanley et al., 2001; Moult et al., 2010; Moult and Harvey, 2011).

**Figure 4:**
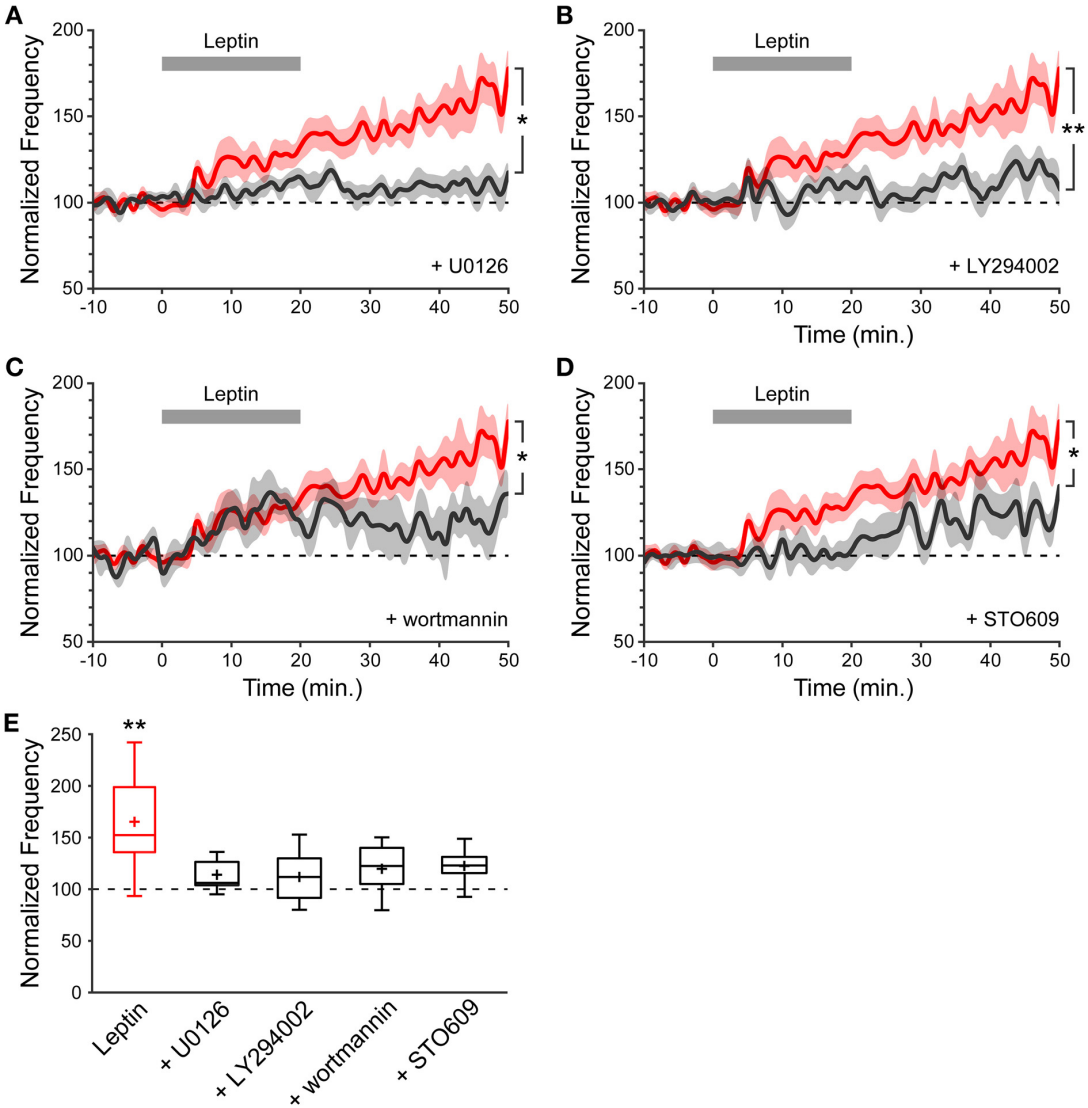
**The leptin-induced potentiation of miniature GABAergic activity involves a postsynaptic signaling pathway comprising MEK1/2, PI3K and CaMKK**. **(A–D)** Normalized frequency of mGABA_A_-PSCs against time (1-minute bins) showing average value (*bold line*) ± SEM (*shaded area*). Leptin was added at *t* = 0−20 min. (*gray bar*). Plotted are Leptin condition (*red*) and conditions (*black*) where leptin was applied in the presence of U0126 **(A)**, LY294002 **(B)**, wortmannin **(C)** or STO609 **(D)** in the recording solution. ^*^p < 0.05, ^**^p < 0.01 vs. Leptin, at the end of the recording (40–50 min.) (unpaired Student's *t*-test). **(E)** Box plots of normalized mGABA_A_-PSC frequency at the end of the recording (40–50 min.). ^*^p < 0.05 vs. baseline (−10−0 min.) (paired Student's *t*-test).

Because leptin treatment of acute hippocampal slices increases pERK immunoreactivity (Figures 2B,C), we first tested the ability of leptin to potentiate the frequency of mGABA_A_-PSCs when U0126 (2 μM), a MEK1/2 inhibitor, was added to the recording solution, and found that U0126 prevented LLP-GABA_A_ (114 ± 6%, *n* = 7, *p* = 0.183, and 0.014 vs. baseline and Leptin resp., Figures 4A,E). We next tested for the possible involvement of PI3K, a key pathway in leptin signaling (Robertson et al., 2008), on leptin-induced LLP-GABA_A_. Addition of the PI3K inhibitors LY294002 (10 μM) or wortmannin (50 nM) to the recording solution comparably prevented the leptin-induced LLP-GABA_A_ (LY294002: 112 ± 8%, *n* = 10, *p* = 0.085, and 0.005 vs. baseline and Leptin resp., Figures 4B,E; wortmannin: 118 ± 11%, *n* = 6, *p* = 0.143, and 0.039 vs. baseline and Leptin resp., Figures 4C,E). Finally, in a recent study we identified a novel signaling cascade mediating the action of leptin through the LepRb and the calcium-calmodulin kinase (CaMK) pathway (Dhar et al., 2014a). We thus sought to determine whether CaMKK could also play a role in leptin-induced LLP-GABA_A_. To this end, STO609 (10 μM), a selective inhibitor of CaMKK (Wayman et al., 2008) was added to the recording solution. We found that the leptin-induced LLP-GABA_A_ was significantly reduced in STO609-loaded neurons (121 ± 7%, *n* = 7, *p* = 0.049, and 0.029 vs. baseline and Leptin resp., Figures 4D,E).

Together, these pharmacological experiments suggest that the potentiation of miniature GABAergic activity following leptin application is transduced by postsynaptic ERK, PI3K and CaMKK. Remarkably, this locus of transduction is similar to the requirement for a postsynaptic rise in intracellular Ca^2+^ through IP_3_-induced Ca^2+^ release that we also observed.

### Leptin enhances the number of functional GABAergic synapses

Results obtained with pharmacological manipulations show that the induction of LLP-GABA_A_relies on the activation of postsynaptic signaling mechanisms. As a next step we explored whether the expression of the leptin-induced LLP-GABA_A_ may be presynaptic and/or postsynaptic. The observation that leptin increases the frequency but not amplitude of mGABA_A_-PSCs suggests that LLP-GABA_A_ is not expressed as a uniform increase in postsynaptic GABA_A_ receptor (GABA_A_R) number or function. Accordingly, peak-scaled variance analysis of mGABA_A_-PSCs revealed that unitary conductance and number of GABA_A_R channels open at the peak of miniature synaptic currents were not affected by leptin application. Unitary conductance ranged from 26.2 ± 2.8 pS to 25.0 ± 2.4 pS (baseline vs. Leptin, *n* = 11, *p* = 0.547; data not shown) while the number of channels ranged from 22 ± 4 to 25 ± 3 (*p* = 0.439). These results indicate that LLP-GABA_A_ cannot be attributed to uniform modifications of postsynaptic GABA_A_R number or function.

Hence leptin-induced LLP-GABA_A_ may be expressed as a modification in GABA release probability and/or in the number of functional GABAergic synapses. To address these possibilities we investigated the effect of leptin on evoked postsynaptic currents. Delivering pairs of identical electrical stimuli at an interval of 50–150 ms in CA3 *stratum radiatum*, we recorded pairs of monosynaptic evoked GABA_A_-PSCs (eGABA_A_-PSCs) from CA3 pyramidal neurons in the presence of NBQX (5 μM) and D-APV (40 μM) at a holding potential of −70 mV. The paired-pulse ratio (PPR) and the coefficient of variation (CV) of eGABA_A_-PSC amplitude were used to assess the site of expression of synaptic plasticity (Faber and Korn, 1991; Manabe et al., 1993).

**Figure 5:**
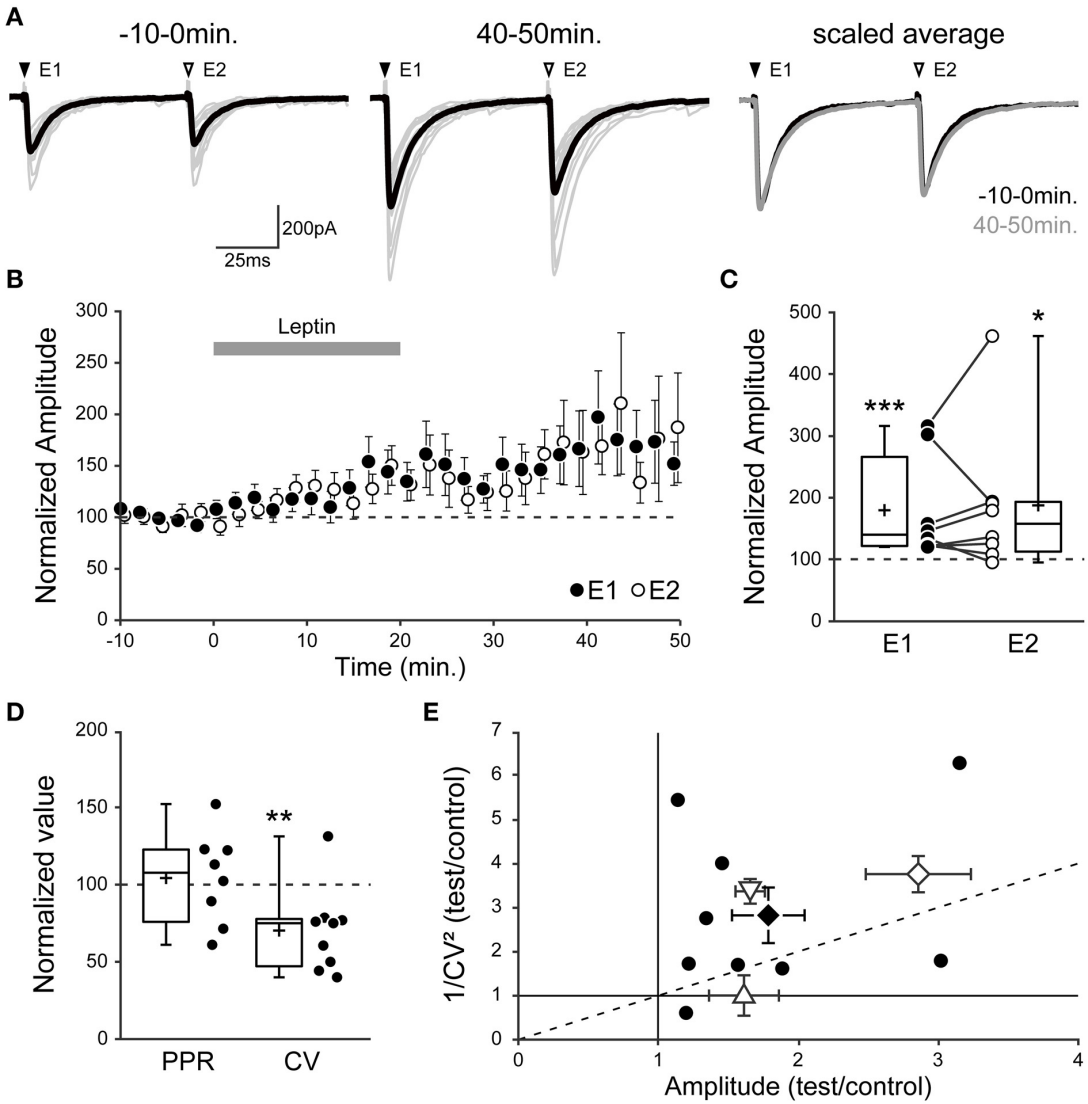
**Leptin enhances the number of functional GABAergic synapses. (A–C)** Evoked GABA_A_ receptor-mediated postsynaptic currents (eGABA_A_-PSCs) were recorded from P1-7 CA3 pyramidal neurons following pairs of identical stimuli at a frequency of 0.01 Hz in CA3 *stratum radiatum*. (**A**, left) Individual traces (gray) and average trace (black) are shown before (−10–0 min) and after (40–50 min) leptin application (100 nM). E1 and E2, evoked currents 1 and 2. (**A**, right) Scaled average traces before vs. after leptin application are superimposed. **(B)** Normalized amplitude of eGABA_A_-PSCs against time (1-min bins) showing average ± s.e.m. Paired data points represent pairs of evoked currents 1 (E1, black circles) and 2 (E2, open circles). **(C)** Box plots and individual recordings of normalized eGABA_A_-PSC amplitude after leptin application (40–50 min) normalized to before leptin application (−10–0 min) for evoked currents 1 (E1, black circles) and 2 (E2, open circles). ^*^p < 0.05, ^***^p < 0.001 vs. before leptin application (paired Student's *t*-test). **(D)** Box plots show normalized values of the paired-pulse ratio (PPR, E2/E1 amplitude) and coefficient of variation (CV, ratio of s.d. over average of E1 amplitude) after leptin application. ^**^p < 0.01 vs. before leptin application (paired Student's *t*-test). **(E)** Scatter plot showing the eGABA_A_-PSC 1/CV^2^ ratio after (“test,” 40–50 min) vs. before (“control,” −10–0 min) leptin application plotted against the mean eGABA_A_-PSC amplitude after (test) vs. before (control) leptin application. Black circles show individual data points, a black diamond represents average ± s.e.m. In three other series of control experiments, the “test” condition was a postsynaptic increase in GABA driving force (point-up triangle), a presynaptic increase in GABA release (point-down triangle) or an increase in the number of recruited synapses (open diamond). Note that the postsynaptic manipulation causes no change in CV, whereas the presynaptic manipulation and a modification in the number of recruited synapses cause a change in CV.

In previous studies we have shown that these parameters can be used in the developing rat hippocampus to assess the site of expression of a depression in eGABA_A_-PSC amplitude (Caillard et al., 1998, 1999; Tosetti et al., 2005). Here we further validated this approach in the case of a potentiation of eGABA_A_-PSC amplitude, through manipulations with a controlled site of expression: 1° a postsynaptic increase in GABA driving force (Figure 5E *point-up triangle*), by hyperpolarizing the recorded neuron, increased eGABA_A_-PSC amplitude (123 ± 15 pA to 193 ± 14 pA, *n* = 4, *p* = 0.01) with no change in PPR (Caillard et al., 1998) nor CV (0.43 ± 0.04 to 0.39 ± 0.01, *p* = 0.4); 2° a presynaptic increase of GABA release (Figure 5E *point-down triangle*), by increasing the extracellular Ca^2+^/Mg^2+^ ratio, increased eGABA_A_-PSC amplitude (184 ± 32 pA to 297 ± 46 pA, *n* = 3), increased the PPR (Caillard et al., 1998) and decreased the CV (0.42 ± 0.02 to 0.24 ± 0.02); and 3° an increase in the number of recruited synapses (Figure 5E *open diamond*), by increasing the stimulus intensity, increased eGABA_A_-PSC amplitude (96 ± 16 pA to 233 ± 13 pA, *n* = 5, *p* = 0.02) with no change in PPR (0.75 ± 0.09 to 0.90 ± 0.05, *p* = 0.15) and decreased the CV (0.53 ± 0.07 to 0.27 ± 0.03, *p* = 0.008). Therefore, the PPR and CV are indicative of the site of expression of GABAergic synaptic plasticity in the developing rat hippocampus. Specifically, a change in PPR reflects presynaptic modifications in GABA release probability, whereas a change in CV reflects presynaptic modifications in GABA release probability and/or modifications in the number of functional GABAergic synapses.

Using this method we measured the effect of leptin by comparing the PPR and CV of eGABA_A_-PSCs before (“control,” −10–0 min) and after (“test,” 40–50 min) leptin application, and observed three possible outcomes in recorded neurons. In 10 out of 28 neurons (36%), leptin had no effect on eGABA_A_-PSC amplitude (102 ± 4% of control, *p* = 0.510; PPR, 1.03 ± 0.06 to 0.99 ± 0.06; CV, 0.30 ± 0.03 to 0.33 ± 0.03; data not shown). In another 9 neurons (32%) leptin led to a depression of eGABA_A_-PSC amplitude (54 ± 6% of control, *p* < 0.001; PPR, 0.84 ± 0.04 to 1.02 ± 0.14; CV, 0.28 ± 0.05 to 0.44 ± 0.11). Finally, in 9 out of 28 neurons (32%) leptin led to a potentiation of eGABA_A_-PSC amplitude (179 ± 26% of control, *p* < 0.001, Figures 5A,B). We focused on the latter outcome in order to investigate the potentiating effect of leptin on GABAergic plasticity. In this group the amplitude of the second eGABA_A_-PSC increased in the same proportion to that of the first event (187 ± 42% of control, *p* = 0.012, Figures 5A–C), thus leaving the PPR unchanged after leptin application (from 0.90 ± 0.08 to 0.89 ± 0.05, *p* = 0.229, Figure 5D). Nevertheless, the increase in eGABA_A_-PSC amplitude was associated with a decrease in CV (from 0.33 ± 0.04 to 0.21 ± 0.02, *p* = 0.01, Figure 5D). As a visual summary, the 1/CV^2^ ratio in test vs. control conditions was plotted against the ratio of eGABA_A_-PSC amplitudes (Manabe et al., 1993). Most recorded neurons in which leptin induced a potentiation of eGABA_A_-PSC amplitude were located in the same region as the control manipulation in which the number of recruited synapses was increased (Figure 5E). Finally, to gain further insight into the site of expression of leptin-induced LLP-GABA_A_ we evaluated the effect of leptin on postsynaptic currents evoked by bath application of the GABA_A_R agonist isoguvacine (3 μM) in the presence of TTX (1 μM). Leptin (100 nM) produced a non-significant and reversible decrease of isoguvacine-induced inward currents (−19.8 ± 8.7%, *p* = 0.2, *n* = 5, data not shown).

Overall, these data indicate that leptin-induced LLP-GABA_A_ does not arise from a uniform postsynaptic change in the number or properties of GABA_A_Rs, but rather from an increase in the number of functional GABAergic synapses.

### GABAergic synaptic transmission is altered in leptin-deficient mice

**Figure 6:**
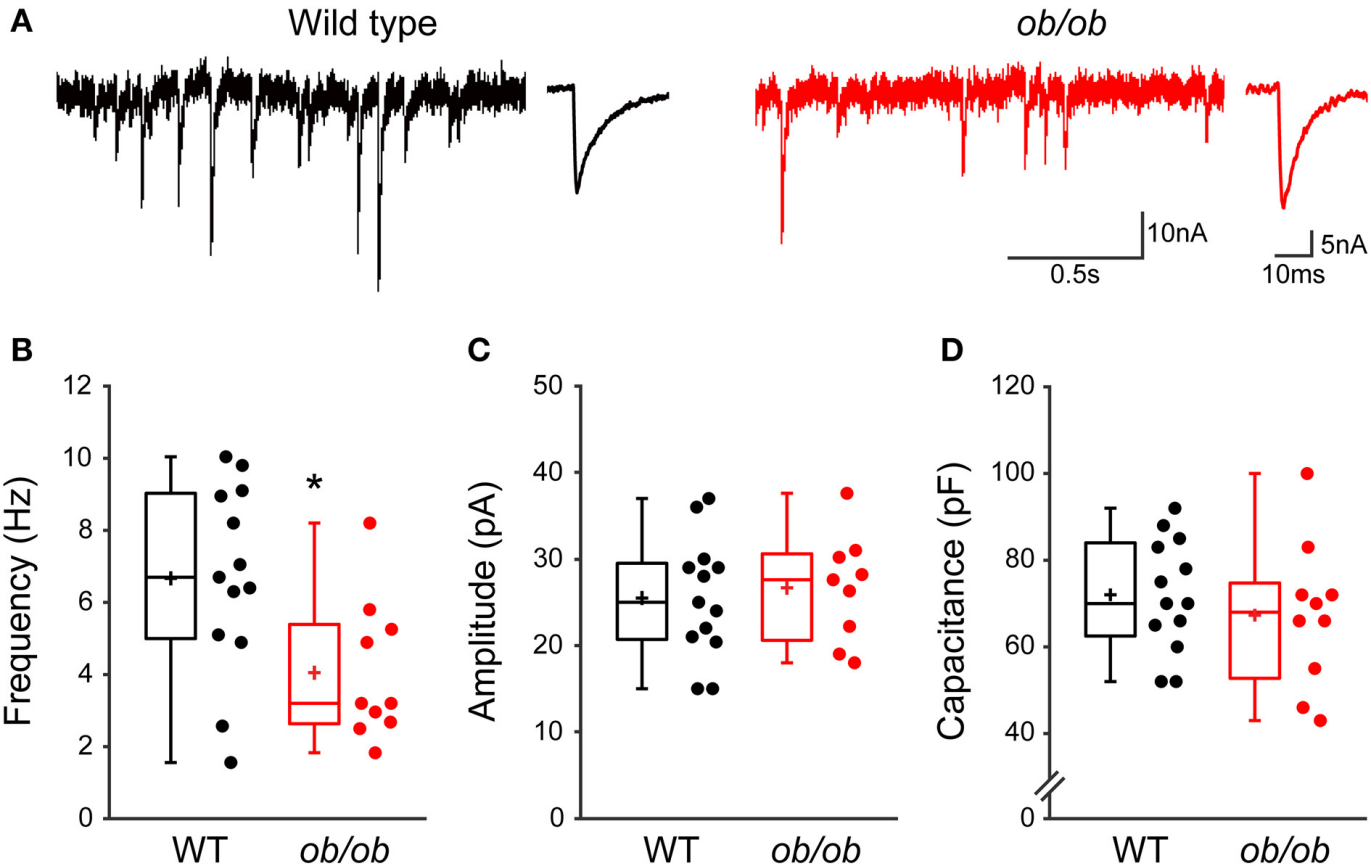
**Miniature GABAergic synaptic activity is altered in leptin-deficient mice. (A)** Acute hippocampal slices were prepared from P10 wild type mice or *ob/ob* mutant littermates and mGABA_A_-PSCs were recorded from CA3 pyramidal neurons. Electrophysiological traces of two representative cells show basal mGABA_A_-PSCs from wild type (black traces) and *ob/ob* (red traces) conditions. For each condition: left, recording; right, averaged current. **(B–D)** Box plots show comparison of frequency **(B)**, amplitude **(C)** and capacitance **(D)** of mGABA_A_-PSCs in wild type (WT) and *ob/ob* conditions. ^*^p < 0.05 vs. WT (unpaired Student's *t*-test).

Our results open the possibility that leptin contributes to the development of GABAergic circuitry in the hippocampus. Seeking to confirm the corollary that chronic leptin deficiency leads to impaired development of GABAergic transmission, we recorded mGABA_A_-PSCs from hippocampal CA3 pyramidal cells of wild type (WT) and leptin-deficient (*ob/ob*) mice at P10, i.e., during the surge of circulating leptin observed in the mouse (Ahima et al., 1998). We observed a selective reduction in the frequency of mGABA_A_-PSCs by 1.61-fold in *ob/ob* compared to WT mice (6.6 ± 0.7 Hz, *n* = 13 in WT vs. 4.1 ± 0.6 Hz, *n* = 10 in *ob/ob*, *p* = 0.016, Figures 6A,B), while other properties of mGABA_A_-PSCs yielded similar values in both genotypes. Thus, the amplitude (26.6 ± 2.1 pA in WT vs. 25.5 ± 1.9 pA in *ob/ob, p* = 0.68, Figure 6C), coefficient of variation (0.75 ± 0.16 in WT vs. 0.58 ± 0.04 in *ob/ob*, *p* = 0.24; data not shown) and kinetic properties (rise and decay times were respectively 1.4 ± 0.3 ms and 8.3 ± 1.8 ms in WT vs. 1.2 ± 0.2 ms and 8.1 ± 0.8 ms in *ob/ob, p* = 0.7 and 0.9) of mGABA_A_-PSCs were unchanged. We also compared the membrane capacitance of recorded neurons, an indicator of cell size (Colin-Le Brun et al., 2004). The capacitance was not different between WT mice and *ob/ob* littermates (72 ± 4 pF vs. 67 ± 5 pF, *p* = 0.45, Figure 6D), suggesting that the gross morphological development of target CA3 pyramidal cells was not affected. Thus, the development of GABAergic synapses is impaired in P10 mice lacking leptin expression, supporting a contribution of leptin to the development of the hippocampal GABAergic circuitry.

**Figure 7:**
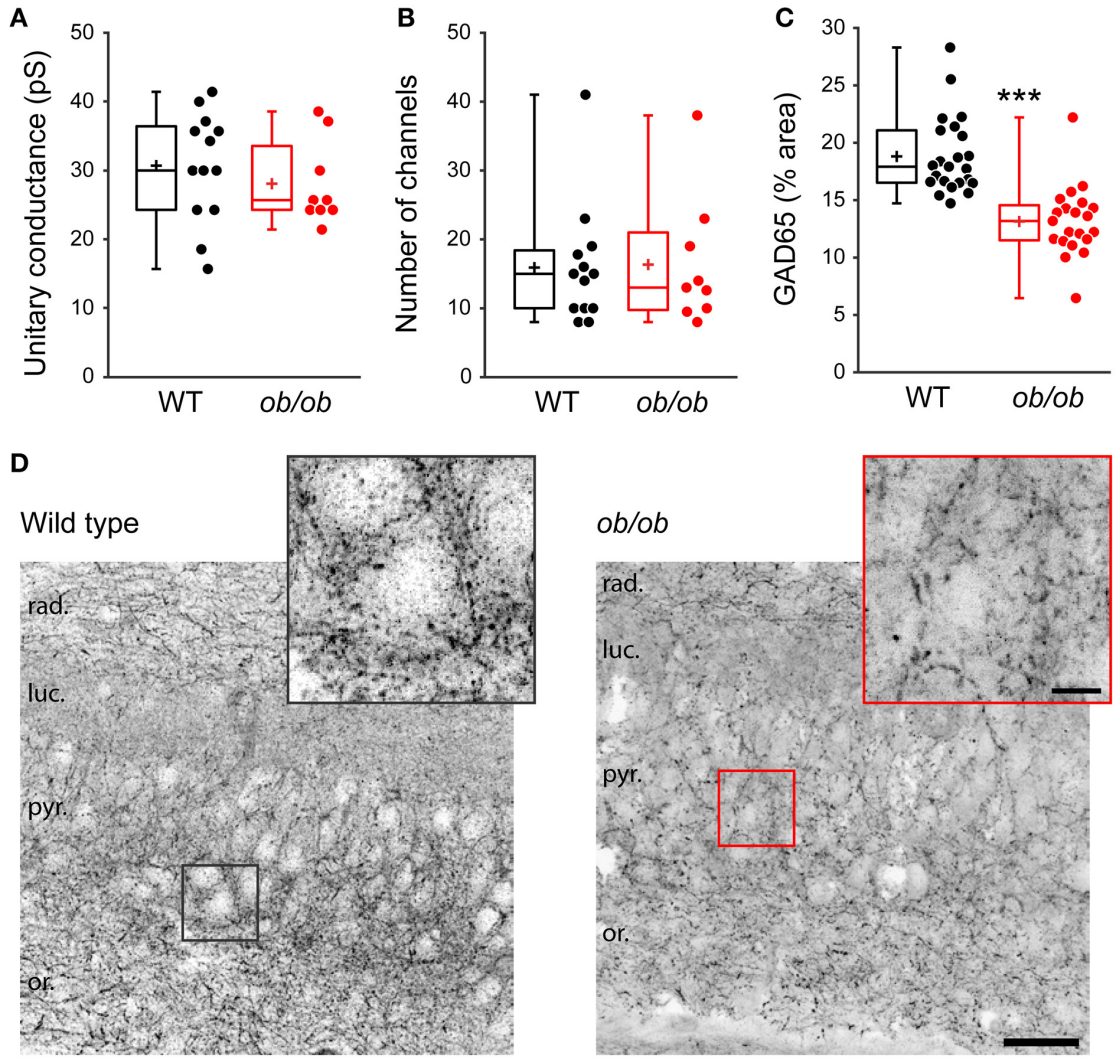
**Properties of individual postsynaptic GABA_A_R channels are unaltered but GABAergic terminals are reduced in leptin-deficient mice**. **(A,B)** Peak-scaled variance analysis was performed on patch clamp recordings of P10 wild type (WT) and *ob/ob* CA3 pyramidal neurons, from which the mean unitary conductance **(A)** and number of postsynaptic channels **(B)** open at the peak of mGABA_A_-PSCs were deduced and are shown as box plots. **(C,D)** The brain of P14 WT and *ob/ob* mice was retrieved, the hippocampus was sectioned and immunostained for glutamic acid decarboxylase 65 (GAD65). **(C)** Box plots of GAD65 immunoreactivity in WT vs. *ob/ob* mice. ^***^p < 0.001 (unpaired Student's *t*-test). **(D)** Representative optical sections show GAD65 immunoreactivity in CA3 of WT and *ob/ob* mice. Insets are close-ups of framed areas in the main images, centered on putative CA3 pyramidal cell somata. Abbreviations: rad., *stratum radiatum*; luc., *stratum lucidum*; pyr., *stratum pyramidale*; or., *stratum oriens*. Scale bars, 50 μm (main image) and 10 μm (inset).

Finally, we asked whether the deficit in GABAergic synaptic transmission in *ob/ob* mice relies on presynaptic and/or postsynaptic modifications. Peak-scaled variance analysis of mGABA_A_-PSCs revealed no significant difference between WT and *ob/ob* mice in unitary conductance (30.5 ± 8.0 pS, *n* = 13 in WT vs. 27.9 ± 6.1 pS, *n* = 9 in *ob/ob, p* = 0.417, Figure 7A) or number of GABA_A_R channels (16 ± 9 in both groups, *p* = 0.912, Figure 7B). Therefore, the alteration of miniature GABAergic activity observed in *ob/ob* mice cannot be attributed to uniform modifications of postsynaptic GABA_A_R channels. To investigate changes in the density of GABAergic terminals we performed immunostaining against glutamic acid decarboxylase 65 (GAD65), the GABA synthetizing enzyme. GAD65 immunoreactivity was decreased by 1.43-fold in the CA3 *stratum pyramidale* of *ob/ob* mice compared to wild type littermate controls (18.8 ± 3.3% area, *n* = 23 in WT vs. 13.1 ± 3.0% area, *n* = 23 in *ob/ob, p* < 0.001, Figures 7C,D). Taken together, these data suggest that the deficit in GABAergic synaptic activity observed in *ob/ob* mice results at least partly from a decrease in the density of proximal GABAergic terminals.

## Discussion

Leptin acts as a neurotrophin during perinatal development of the central nervous system in rodents, and as such is essential for the formation of key neuronal pathways (Bouret, 2010). Leptin has been shown to induce glutamatergic synapse formation and maturation in the hippocampus (Durakoglugil et al., 2005; O'Malley et al., 2007; Moult et al., 2009, 2010; Moult and Harvey, 2011; Dhar et al., 2014b), which expresses leptin receptors at early ages in CA1, CA3 and the dentate gyrus (Mercer et al., 1996; Shanley et al., 2002b; Guo et al., 2012). However, the action of leptin on developing hippocampal GABAergic synapses is not known. Here we show for the first time that leptin enhances GABAergic synaptic transmission in the neonatal rodent hippocampus, at an age when GABA is key to the development of the hippocampal neuronal network by exerting most of the excitatory drive onto pyramidal cells (Ben-Ari et al., 2007). Furthermore, we provide evidence that this potentiation is induced by postsynaptic signaling pathways which require a rise in cytoplasmic calcium released from internal stores, PI3K, CaMKK, and MEK1/2. We also show that leptin potentiates developing GABAergic synaptic activity by increasing the number of functional GABAergic synapses. Finally we report that CA3 pyramidal neurons of leptin-deficient *ob/ob* mice exhibit lower GABAergic activity and a decreased density of presynaptic terminals compared to wild type littermates, suggesting that leptin contributes to the development of the hippocampal GABAergic circuitry *in vivo*.

### Leptin activates a postsynaptic molecular signaling pathway to potentiate developing GABAergic synapses

Previous studies have reported that the long form of the leptin receptor (LepRb) is present and functional at both pre- and postsynaptic sites in the hippocampus (Mercer et al., 1996; Elmquist et al., 1998; Shanley et al., 2002b; Guo et al., 2012). Here we showed that leptin potentiates GABAergic activity at low concentrations (3–20 nM) and confirmed that the newborn rat hippocampus contains LepRb mRNA. Further, we found that exogenous leptin application on acute hippocampus slices increases pERK immunoreactivity in CA3 *stratum pyramidale*, an effect not indirectly mediated by network activity as the latter was blocked. Finally, the observation that chelation of postsynaptic calcium and inhibition of postsynaptic kinases prevented the induction of LLP-GABA_A_ indicates that leptin acts on postsynaptic receptors to potentiate GABAergic synaptic activity. Although a nonspecific action of leptin on other cytokine receptors cannot be ruled out, this body of evidence points to the binding of leptin to LepRb on the postsynaptic cell as the trigger for GABAergic synaptic potentiation. Intriguingly we found that leptin application (100 nM) for 20 min promoted the transcription of LepRb mRNA (data not shown), a result which should be further investigated. Indeed, if the effects of leptin on the hippocampus are confirmed to be mediated by the LepRb, then activation of this receptor could lead to a positive feedback loop in the developing hippocampus.

The precise molecular mechanism through which leptin enhances GABAergic synaptic activity in the developing hippocampus awaits further exploration. Nevertheless, we found that postsynaptic PLCγ activation and IP_3_-induced calcium release from internal stores are required. We also found that PI3K, CaMKK, and ERK likely mediate the potentiation of GABAergic transmission induced by leptin in the developing hippocampus. These results converge with previous studies showing that most, if not all actions of leptin in the hippocampus described so far are mediated by PI3K- and ERK-dependent downstream pathways. Thus, *in vitro* and *in vivo* administration of leptin had no detectable effect on pSTAT3 but increased pERK immunoreactivity in the hippocampus (Walker et al., 2007; Caron et al., 2010; Dhar et al., 2014b). Moreover leptin was found to modulate hippocampal glutamatergic and GABAergic synaptic efficacy and neuronal excitability through PI3K- and/or ERK-dependent mechanisms (Shanley et al., 2001, 2002a,b; O'Malley et al., 2005; Xu et al., 2008; Solovyova et al., 2009; Moult and Harvey, 2011).

How these signaling molecules interact to mediate leptin-induced LLP-GABA_A_ remains to be investigated. Yet, based on the literature and on our results we put forward the following hypotheses. PI3K, one of the signaling molecules commonly activated by the LepRb (Frühbeck, 2006), is known to interact with and activate PLCγ *in vitro* and in several cell types following growth factor stimulation (Bae et al., 1998; Falasca et al., 1998; Rameh et al., 1998). In addition leptin has been reported to trigger a PI3K-dependent activation of PLCγ in hypothalamic POMC neurons (Qiu et al., 2010). Thus, PI3K could mediate PLCγ activation by the LepRb, leading to the production of IP_3_. A modification of the phosphorylation state of IP_3_Rs, possibly via PI3K but independently of PLCγ, could also increase IP_3_R sensitivity to cytoplasmic IP_3_ and thus contribute to the induction of LLP-GABA_A_. Indeed IP_3_Rs are prone to regulation by a number of kinases including the serine/threonine protein kinase Akt, a downstream effector of PI3K (Foskett et al., 2007). The acute rise in cytoplasmic calcium produced by IP_3_R opening would activate the CaMK pathway through activation and autophosphorylation of CaMKK and CaMKI, leading to MEK/ERK phosphorylation/activation (Wayman et al., 2006). Consistent with this hypothesis we have shown that leptin activates CaMKK and CaMKI in dissociated hippocampal cultures (Dhar et al., 2014a). Another possibility is that the PI3K and MAPK pathways, both activated by the LepRb (Frühbeck, 2006) would converge to a single effector requiring both signals to mediate GABAergic synaptic potentiation. The downstream pathway activated by these kinases remains to be explored. Potential targets could include protein synthesis (conceivably under the control of the MEK/ERK/CREB pathway), which is known to support persistent changes in synaptic strength in many forms of synaptic plasticity.

### Leptin-induced potentiation of GABAergic activity is expressed as an increase in the number of functional GABAergic synapses

We found that leptin has no effect on the amplitude of mGABA_A_-PSCs, making a uniform modification of postsynaptic receptor number and/or function implausible. We also found that leptin enhances the frequency of mGABA_A_-PSCs and decreases the CV of eGABA_A_-PSCs, consistent with a presynaptic alteration in GABA release and/or a modification in the number of functional GABAergic synapses recruited. However, whereas manipulating presynaptic GABA release alters the PPR of eGABA_A_-PSCs (Caillard et al., 1999), we observed no modification in the PPR of eGABA_A_-PSCs following leptin application. Taken together, these results argue that the leptin-induced LLP-GABA_A_ is expressed as an increase in the number of functional GABAergic synapses. Such a plasticity mechanism has been reported for GABAergic synapses in the goldfish and in the neonatal rat hippocampus (Charpier et al., 1995; Gubellini et al., 2001). This effect may occur presynaptically by “switching on” previously silent synapses and/or postsynaptically by clustering postsynaptic receptors at functionally weak or silent inhibitory synapses, as has been proposed for glutamatergic synapses (Charpier et al., 1995; Voronin and Cherubini, 2004).

Because postsynaptic signaling pathways are required for leptin to induce LLP-GABA_A_, one target of the postsynaptic signaling cascade unveiled here could be GABA_A_Rs. Indeed membrane expression of GABA_A_Rs is a highly dynamic process and GABA_A_R trafficking is considered to be an effector downstream of extracellular neuropeptide cues (Luscher et al., 2011). Thus, GABA_A_Rs could be recruited to silent postsynaptic sites through inhibition of endocytosis, increased membrane insertion and/or lateral diffusion (Luscher et al., 2011). Interestingly, treatment of hippocampal neuronal cultures with BDNF or insulin have been reported to increase GABA_A_R cell surface expression at postsynaptic sites via a PI3K signaling cascade (Wan et al., 1997; Vetiska et al., 2007; Porcher et al., 2011). Moreover, PI3K and/or ERK activation by the LepRb have been shown to promote membrane insertion of several other proteins impacting electrophysiological properties of hippocampal neurons, including potassium channels, TRPC channels and glutamatergic receptors (O'Malley et al., 2005; Moult et al., 2010; Dhar et al., 2014a).

Here we found a dual effect of leptin on eGABA_A_-PSCs, whereby leptin can lead to a depression or a potentiation of evoked currents. One possible explanation for this could be that the action of leptin relies on the activation of distinct leptin receptors differing in their location and/or downstream effectors, as leptin receptors are present both pre- and postsynaptically in the hippocampus (Shanley et al., 2002b; Solovyova et al., 2009). Moreover, leptin has been reported to have dual effects on the membrane potential of hippocampal neurons. Thus, leptin can either hyperpolarize or depolarize hippocampal neurons through the activation of Ca^2+^-dependent potassium channels or TRPC channels, respectively (Shanley et al., 2002a; Dhar et al., 2014a). Leptin can also cause either a hyperpolarization or a depolarization of different subsets of neurons in the ventral premamillary nucleus of the hypothalamus (Williams et al., 2011). Hence, one could speculate that leptin presynaptically modulates distinct subsets of GABAergic interneurons by decreasing cell excitability and/or modulating Ca^2+^ entry. Differential presynaptic modulation of GABAergic activity is indeed a well described feature of the two major types of perisomatic inhibitory synapses, namely synapses formed by parvalbumin and cholecystokinin basket cells onto hippocampal pyramidal neurons.

The present study complements previous reports of leptin modulating GABAergic synaptic transmission. Acute leptin treatment of hypothalamic slices decreases the frequency of spontaneous and miniature GABA_A_-PSCs onto POMC neurons (Cowley et al., 2001; Munzberg et al., 2007; Vong et al., 2011). Conversely, a marked increase in GABA_A_-PSC frequency is observed in POMC neurons when leptin signaling is chronically impaired in mutant mice (Pinto et al., 2004; Vong et al., 2011). The signaling pathway mediating leptin-dependent GABAergic plasticity in the hypothalamus is not entirely clear, but genetic manipulations indicate that leptin acts on presynaptic GABAergic neurons to reduce GABA release (Vong et al., 2011). Consistent with these findings, LepRbs are located near synapses on the soma and dendrites of hypothalamic GABAergic neurons, suggesting that leptin could act presynaptically to modulate GABAergic synaptic activity in this region (Ha et al., 2013).

In the hippocampus, Solovyova et al. (2009) reported that acute leptin treatment leads to a PI3K-dependent potentiation of miniature GABAergic activity onto hippocampal neurons. However, in contrast to our findings this potentiation was transient and expressed through a uniform increase in number and/or function of postsynaptic GABA_A_Rs. In addition the authors reported that leptin leads to a persistent depression of eGABA_A_-PSCs, whereas we could make this observation in only a subset of stimulated GABAergic synapses. Interestingly this apparent discrepancy could reveal differences in the spatiotemporal pattern of leptin's action, as the two studies were conducted in different hippocampal fields [CA3 (the present study) vs. CA1 (Solovyova et al., 2009)] and in different rat strains (Wistar vs. Sprague-Dawley). Moreover the findings were obtained at P1-7 vs. P13–19, i.e., at stages of hippocampal development which differ in number of GABAergic synapses and, crucially, polarity of GABA_A_-PSCs (Danglot et al., 2006). Accordingly, the modulation of glutamatergic transmission by leptin in the hippocampus has been reported to be developmentally regulated, as leptin potentiates glutamatergic synapses at adult stages but depresses them at younger stages (Moult et al., 2010; Moult and Harvey, 2011). Likewise BDNF, a key neurotrophin for brain development, has been reported to promote the development of GABAergic synaptic activity at early stages but to depress its efficacy later (Gottmann et al., 2009; Kuczewski et al., 2009). Although a clear picture of leptin's action on hippocampal development and function is yet to be drawn, based on these studies and on the present findings one can envisage that leptin signaling modulates the development and plasticity of both glutamatergic and GABAergic types of synaptic transmission in the hippocampus.

### Physiological implication of the contribution of leptin to the development of GABAergic synapses in the hippocampus

Leptin plays a central role in the regulation of energy homeostasis through its action on specific hypothalamic nuclei. Within the hypothalamus, GABAergic neurons are a key target of leptin to regulate feeding behavior and reproductive function (Vong et al., 2011; Zuure et al., 2013). In addition to its regulatory role in the adult hypothalamus, leptin also fulfills an important developmental role during the formation of hypothalamic circuitry (Bouret et al., 2004a,b; Bouret and Simerly, 2007). Importantly the trophic action of leptin during development is long-lasting, as early leptin treatment (P4-P16) of *ob/ob* mice reduces excessive food intake in adults compared to non-treated animals (Bouret et al., 2004b). Likewise, rat neonates (P2-P14) exposed to elevated leptin display an altered pattern of synaptic protein expression in the hippocampus at adult stage (Walker et al., 2007). Here we unveil a novel phenotype in the hippocampus of young *ob/ob* mice, namely reduced miniature activity of GABAergic synapses in CA3 pyramidal neurons and reduced immunostaining of perisomatic GABAergic terminals, suggesting a reduced number of GABAergic synapses impinging on CA3 pyramidal neurons. Considering that in rodents the postnatal leptin surge correlates with the time course of GABAergic synaptogenesis (Devaskar et al., 1997; Rayner et al., 1997; Ahima et al., 1998; Ben-Ari et al., 2007), these results lend support to the proposition that leptin contributes to the development of GABAergic networks beyond the hypothalamus. In future studies it should be interesting to assess the action of leptin on adult hippocampal GABAergic plasticity, and any behavioral relevance it might bear.

GABAergic interneurons are key in regulating neuronal excitability and network oscillation dynamics, and as such play an important role in brain function. Consequently, abnormal levels of GABA or impaired GABAergic efficacy are associated with cognitive deficits and neurological disorders (Brambilla et al., 2003; Charych et al., 2009; Deidda et al., 2014). Leptin deficiency during development also leads to altered cognitive performance and neurological disorders such as major depressive disorder (MDD), while leptin replacement alleviates these symptoms and improves cognitive development (Antonijevic et al., 1998; Kraus et al., 2002; Westling et al., 2004; Esel et al., 2005; Lu et al., 2006; Paz-Filho et al., 2008; Signore et al., 2008; Yamada et al., 2011; Guo et al., 2012). Based on the present findings, one can speculate that altered leptin signaling may compromise the development of GABAergic transmission, to at least partly underlie cognitive deficits and neurological disorders associated with leptin deficiency or leptin resistance.

### Conflict of interest statement

The authors declare that the research was conducted in the absence of any commercial or financial relationships that could be construed as a potential conflict of interest.
